# Optimization of exogenous carbohydrases supplemented in broiler diets using *in vitro* simulated gastrointestinal digestion and response surface methodology

**DOI:** 10.1371/journal.pone.0259865

**Published:** 2021-11-15

**Authors:** Yang Liu, Shengli Liu, Guitao Jiang, Qiuzhong Dai

**Affiliations:** 1 Hunan Institute of Animal Husbandry and Veterinary Medicine, Changsha, China; 2 College of Animal Science and Technology, Hunan Agriculture University, Changsha, China; 3 Shandong Lonct Enzymes Co., Ltd., Linyi, China; University of Life Sciences in Lublin, POLAND

## Abstract

The present study aimed to explore the optimal zymogram of combination of 6 carbohydrases (glucoamylase, pullulanase, maltase, thermostable α-amylase, medium temperature α-amylase, and cold-active α-amylase) supplemented in corn-soybean based diet of broilers aged 1 to 3 wk for the maximum starch digestibility, by using *in vitro* simulated gastrointestinal digestion and response surface method. The third generation of simulated monogastric animal digestion system was used for *in vitro* digestion experiment. By using single factor completely random design, the optimal supplement levels of single carbohydras were determined by the reducing sugar release amount and improved dry matter digestibility, which were the parameters representing the starch digestibility of the diet. Additionally, Box-Behnken response surface method was used to predict the optimal combination of 6 carbohydrases. The results showed that the optimistic zymogram of 6 carbohydrases in corn-soybean based diet for broilers aged 1 to 3 wk were 297.39 U/g glucoamylase, 549.72 U/g pullulanase, 3.01 U/g maltase, 1,455.73 U/g thermostable α-amylase, 278.64 U/g medium temperature α-amylase, and 1,985.97 U/g cold-active α-amylase, and the associated reduced sugar release amount and improved dry matter digestibility were 215.98 mg/g, and 6.23%, respectively. Furthermore, we conducted *in vitro* digestion experiments with diets supplemented with the predicted optimistic zymogram and found that the experimental reduced sugar release amount and improved dry matter digestibility were 219.26 mg/g and 6.31% respectively, whose errors to the predicted optimistic reducing sugar release amount and the improved dry matter digestibility were 1.05% and 1.02%. To sum up, the predicted optimal zymogram of 6 carbohydrases in the present study were capable to improve the starch digestibility in diet for broilers aged 1 to 3 wk, which were represented by increased reduced sugar release amount and improved dry matter digestibility.

## Introduction

Starch is the most important energy source in feed ingredients for broilers [1]. It is mainly consisted by amylopectin and amylose, of which amylopectin has α-1-4 glucose chains with frequent branches due to α-1-6 bonds, whereas amylose only has very few branches [2]. Among cereal sources, starch granules in native status vary in regard to size, shape and even molecular architecture, which lead to the inconsistences in starch digestion for many animal species [3]. It was reported that highly organized structure could pose a challenge for starch digestion, and Zhang et al. [4] stated that the digestibility of starch was related to the ordered structure of alternating crystalline and amorphous layers in the starch granule. Additionally, the physical barriers in cell walls of feed ingredients could restrict enzyme access to the substrates, and reduced the starch digestibility of the animals [5]. Although starch digestibility was relatively high in broilers, certain proportion of the dietary starch was still wasted in the small intestine [6]. Besides, under commercial condition, broilers are frequently fed different feed ingredients and forms from the starter phrase (1 to 21 d) to the growing phrase (22 d to slaughter) considering the lower nutritional digestibility and more feed waste rate at the starter phrase [7]. Therefore, there have been increased interests in improving the utilization of starch in the poultry feed, especially for the diets for birds aged 1 to 21 d. Nowadays, the intensive farming system for broilers requires special processing for the feeds, such as grinding, blending, and pelleting. These processes would break the granular structure of the native starch, and facilitation of the starch digestibility for broilers was expected. However, no changes or inconsistent results in apparent starch digestibility in broilers were reported in previous papers [8–10]. The use of exogenous carbohydrases was another strategy to improve the starch digestibility of the broilers. Carbohydrases are a group of enzymes that degrade the complex starch granules, including but not limited to glucoamylase, amylase, pullulanase and maltase. Multiple reports stated that the addition of exogenous carbohydrases in corn [11], wheat [12], and sorghum-based diet [13] could improve the apparent digestibility of broiler chicken.

However, it was challenging to optimize the combination of exogenous carbohydrases by using broiler models directly, due to the individual variations, long-lasting experimental times, extreme costs, and intensive label requirements. *In vitro* simulated gastrointestinal digestion method represented a possible alternation to assess the integrated effects of various enzymes combination along the poultry digestive tract. In the procedure of using *in vitro* simulated gastrointestinal digestion method, simulated small intestinal fluid with amylase, trypsin, and chymotrypsin reagents was made equal activities to those of *in vivo* small intestinal fluid, and an automatic equipment for simulating the process of gizzard-intestinal digestion were imitated as well [14]. A good repeatability and additivity of *in vitro* digestible energy, and accuracy of predicted metabolic energy of feed were reported previously [15,16], which indicated that the method was promising in predict the energetic value of feed for poultry. Therefore, the objective of the present study was to optimize the zymogram of 6 exogenous carbohydrases, including glucoamylase, pullulanase, maltase, thermostable α-amylase, medium temperature α-amylase, and cold-active α-amylase, for the optimistic starch digestibility in corn-soybean based diet for broilers using *in vitro* simulated gastrointestinal digestion method.

## Materials and methods

### Materials

The exogenous carbohydrases used in the present study were manufactured via microbial engineering, that were kindly provided by Shandong Lonct Enzymes Co., Ltd. (Linyi, Shandong, China). The activities of the glucoamylase, pullulanase, maltase, thermostable α-amylase, medium temperature α-amylase, and cold-active α-amylase were 200,000, 8,000, 1,000, 40,000, 5,000, and 20,000 U/ml, respectively. The pepsase and amylase, which were purchased from Sigma Aldrich Co. (St. Louis, MO, USA), and trypsase and chymase, which were purchased from Amresco Co. (Houston, TX, USA), were used in the simulated small intestinal fluid. The water used in the present study was deionized with conductivity less than 0.5 us/cm and pH between 6.8 and 7.2. And the *in vitro* digestion experiments were conducted in the third generation of simulated monogastric animal digestion system (SDS-Ⅲ, Zhongben Intelligent Technology Development Co., Ltd., Changsha, Hunan, China). The preparations of the digestive fluid, as well as the machine settings were followed by Zhao’s descriptions [14].

The composition of corn-soybean based diets for broilers at 1 to 3 wk was followed arbor acres plus standard, which was showed in Table 1.

**Table 1:** Composition and nutrient levels of basal diets (air-dry basis) for broilers aged 1 to 3 wks.

Items	Content %	Nutrient levels ^2^ %	
Corn	49.59	ME/(MJ/kg)	12.26
Soybean meal (43%)	26.30	CP	22.00
Soybean oil	2.00	EE	4.88
Wheat middling	4.80	DLys	1.14
Limestone	1.42	DMet	0.59
Distiller’s dried grain with soluble	5.00	DThr	0.75
CaHPO_4_	1.33	DMet+DCys	0.88
Peanut meal (47%)	3.00	Ca	0.98
Cottonseed meal (46%)	2.80	AP	0.49
Corn protein meal (60%)	2.00	TP	0.72
*L*-Thr	0.08		
*L*-Lys	0.50		
*DL-*Met	0.18		
Premix ^1^	1.00		
Total	100.00		

^1^The premix provided the following per kg of diets: VA 10,000.00 IU, VB_1_ 5.60 mg, VB_2_ 11.00 mg, VB_6_ 8.00 mg, VB_12_ 0.02 mg, VD_3_ 3,000.00 IU, VE 40.00 IU, VK_3_ 2.50 mg, biotin 0.15 mg, folic acid 2.00 mg, D-pantothenic acid 32.00 mg, nicotinic acid 60.00 mg, antioxidant 100.00 mg, Cu (as copper sulfate) 10.00 mg, Fe (as ferrous sulfate) 80.00 mg, Mn (as manganese sulfate) 60.00 mg, Zn (as zinc sulfate) 35.00 mg, I (as potassium iodide) 0.42 mg, Se (as sodium selenite) 0.30 mg.

^2^Nutrient levels were calculated values.

### Experiment design

#### *In vitro* simulated digestion of diets supplemented with exogenous carbohydrase individual

The tested exogenous carbohydrases were arranged with 5 supplement levels in the diets and carried out in the *in vitro* simulated digestion experiments for the reducing sugar release amount (**RSA**) and the improved dry matter digestibility (**DMD**), which were calculated using Eqs 1 and 2, respectively.

$$DMD(\%)=(M_{1}-M_{2})/M_{1}\times 100$$ (1)

where *DMD* is the dry matter digestibility (%), *M*_*1*_ is the dry matter weight of the sample before digestion (g), *M*_*2*_ is the dry matter weight of the undigested sample after digestion (g).

$$RSA\;(mg/g)=\left[\left(a\times OD_{1}+b\right)\times D\times V-\left(a\times OD_{2}+b\right)\times 17.6\right]/\left(w\times DM\right)$$ (2)

where *RSA* is the sugar release amount of the feed sample in in vitro digestion experiment, *a* and b are regression coefficient of the standard curve, *OD*_*1*_ is the OD value of the tested sample, *OD*_*2*_ is the OD value of the empty sample, *D* is the dilution ratio of the sample, *V* is the experiment volume, *w* is the weight of the tested sample, *DM* is the dry matter weight of the tested sample.

The detailed design for the supplement level for each carbohydrase and tested parameters was listed in Table 2. Each supplement level had 5 repeated samples. And the levels of individual carbohydrase achieving the optimistic RSA and improved DMD were calculated separately.

**Table 2:** Supplemental levels of the 6 enzymes.

Treatments	Supplement level U/g					
	Glucoamylase	Pullulanase	Maltase	thermostable α-amylase	Medium temperature α-amylase	Cold-active α-amylase
RSA						
1	10.00	10.00	0.16	80.00	10.63	64.00
2	20.00	20.00	0.31	160.00	21.25	128.00
3	40.00	40.00	0.63	320.00	42.50	256.00
4	80.00	80.00	1.25	640.00	85.00	512.00
5	160.00	160.00	2.50	1,280.00	170.00	1,024.00
6	320.00	320.00	5.00	2,560.00	340.00	2,048.00
Improved DMD						
7	12.50	25.00	0.16	68.75	10.00	68.75
8	25.00	50.00	0.31	137.50	20.00	137.50
9	50.00	100.00	0.63	275.00	40.00	275.00
10	100.00	200.00	1.25	550.00	80.00	550.00
11	200.00	400.00	2.50	1,100.00	160.00	1,100.00
12	400.00	800.00	5.00	2,200.00	320.00	2,200.00

RSA: Reducing sugar release amount; DMD: Dry matter digestibility.

#### Optimization of combined exogenous carbohydrases

Response surface methodology with a three-level, six-variable Box-Behnken design was employed, requiring 54 different experiments for the optimization of the starch degrading zymogram. The independent variables and their levels were showed in Table 3. The RSA (*y*_*1*_) and improved DMD (*y*_*2*_) were chosen as the response variables of the design experiments. Second-order polynomial Eq 3, which contains all interaction terms, was utilized to calculate the predicted RSA and improved DMD.

$$y=b_{0}+\sum_{i=1}^{6}b_{i}x_{i}+\sum_{i=1}^{6}b_{ii}x_{i}^{2}+\sum_{i<j}^{6}b_{ij}x_{i}x_{j}$$ (3)

where *y* is the response variable, *b*_*0*_ is the value for the fixed response at the central point of the experiment, b_i_ is the linear effect, *b*_*ii*_ is the quadratic effect, *b*_*ij*_ is the interaction effect, and *x*_*i*_ and *x*_*j*_ are independent variables.

**Table 3:** Independent variables and their ranges used for zymogram optimization in the Box Behnken design.

		Coded and actual levels U/g		
Independent variable	Factor	-1	0	1
Glucoamylase	*x*_*1*_	269.65	284.83	300.00
Pullulanase	*x*_*2*_	532.56	541.28	550.00
Maltase	*x*_*3*_	2.00	2.60	3.20
Thermostable α-amylase	*x*_*4*_	1,385.71	1,442.86	1,500.00
Medium temperature α-amylase	*x*_*5*_	269.50	277.25	285.00
Cold-active α-amylase	*x*_*6*_	1,965.00	1,982.50	2,000.00

The Design Expert program (Version 8.0.6) was chosen for analyzing the experimental results. Finally, the zymograms of carbohydrases for starch degradation were tested in SDS-Ⅲ to compare with the predicted response variables.

#### Statistical analysis

The level of significance was set a *P* < 0.05. Regression analysis were conducted in Statistical Package for the Social Sciences 19.0 (IMB, Armonk, New York). Box-Behnken design was employed to investigate the response of 6 exogenous carbohydrases. Experimental design, model calculation, and graph drawing were performed using Design Expert Software (Version 8.0.6, Stat-Ease Inc., Minneapolis, USA).

## Results

### Optimization of exogenous carbohydrase individual in diet via *in vitro* simulated digestion method

The RSA and improved DMD of the diets for broilers aged 1 to 3 wk supplemented with tested exogenous carbohydrase individual were showed in Table 4. Significant quadratic relationships were found between the supplement levels of exogenous carbohydrase individuals and RSA as well as improved DMD in diets (*P* < 0.01). As the supplement levels of glucoamylase, medium temperature α-amylase increased within the tested range, the RSA and improved DMD increased. Additionally, as the supplement level of pullulanase increased within the tested range, the RSA in the diet increased, but the improved DMD increased first then decreased. And when the supplement level of pullulanase were at 400 U/g, the improved DMD reached its maximum of 1.29%. Moreover, as the supplement levels of maltase and thermostable α-amylase increased, the RSA and improved DMD of the diets were first increased then decreased. When the supplement levels of maltase and thermostable α-amylase were at 1.25 U/g and 1280 U/g, the RSA reached the highest of 8.88 mg/g and 13.3 mg/g, respectively. When maltase and thermostable α-amylase were at 2.5 U/g and 1100 U/g, the improved DMD reached the highest of 0.45% and 0.54%, respectively. Finally, as the supplement level of cold-active α-amylase increased, the improved DMD in diets increased, but the RSA increased first then decreased. And RSA reached its maximum of 29.33 mg/g when the supplement level of cold-active α-amylase were 1024 U/g.

**Table 4:** Effects of supplementation of exogenous carbohydrase on the reducing sugar release rate and the improved dry matter digestibility of diets for broilers aged 1 to 3 wk.

Item	Content							Quadratic regression equation	*P* value	Optimal supplement level, U/g	Maximun RS, mg/g or improved DMD, %
Glucoamylase	Supplement level, U/g	10.00	20.00	40.00	80.00	160.00	320.00	y = -0.001x^2^ + 0.539x + 2.786	<0.01	269.65	75.50
	RSA, mg/g	6.87	15.71	22.47	37.91	65.91	77.85				
Pullulanase	Supplement level, U/g	10.00	20.00	40.00	80.00	160.00	320.00	y = -0.000001x^2^ + 0.106x - 0.998	<0.01	532.50	27.36
	RSA, mg/g	0.12	1.22	3.16	6.29	13.70	22.27				
Maltase	Supplement level, U/g	0.16	0.31	0.63	1.25	2.50	5.00	y = -2.092x^2^ + 8.382x + 1.187	<0.01	2.00	9.58
	RSA, mg/g	1.90	3.50	6.21	8.88	8.53	-9.10				
Thermostable α-amylase	Supplement level, U/g	80.00	160.00	320.00	640.00	1,280.00	2,560.00	y = -0.000007x^2^ + 0.019x - 0.945	<0.01	1,385.71	12.50
	RSA, mg/g	0.99	1.95	4.16	8.57	13.30	5.10				
Medium temperature α-amylase	Supplement level, U/g	10.63	21.25	42.50	85.00	170.00	340.00	y = -0.0002x^2^ + 0.108x + 1.431	<0.01	269.50	15.96
	RSA, mg/g	2.71	3.93	5.80	8.33	15.91	19.69				
Cold-active α-amylase	Supplement level, U/g	64.00	128.00	256.00	512.00	1,024.00	2,048.00	y = -0.00001x^2^ + 0.039x + 3.597	<0.01	1,965.00	42.21
	RSA, mg/g	5.15	7.96	14.68	20.08	29.33	28.18				
Glucoamylase	Supplement level, U/g	12.50	25.00	50.00	100.00	200.00	400.00	y = -0.000004x^2^ + 0.002x + 0.217	<0.01	300.00	0.58
	Improved DMD, %	0.23	0.28	0.32	0.43	0.53	0.57				
Pullulanase	Supplement level, U/g	25.00	50.00	100.00	200.00	400.00	800.00	y = -0.000003x^2^ + 0.003x + 0.408	<0.01	550.00	1.32
	Improved DMD, %	0.51	0.59	0.67	0.91	1.29	1.16				
Maltase	Supplement level, U/g	0.16	0.31	0.63	1.25	2.50	5.00	y = -0.024x^2^ + 0.152x + 0.213	<0.01	3.20	0.46
	Improved DMD, %	0.23	0.27	0.30	0.35	0.45	0.38				
Thermostable α-amylase	Supplement level, U/g	68.75	137.50	275.00	550.00	1,100.00	2,200.00	y = -0.0000002x^2^ + 0.0006x + 0.16	<0.01	1,500.00	0.61
	Improved DMD, %	0.19	0.23	0.32	0.43	0.54	0.40				
Medium temperature α-amylase	Supplement level, U/g	10.00	20.00	40.00	80.00	160.00	320.00	y = -0.00002x^2^ + 0.011x + 0.131	<0.01	285.00	1.76
	Improved DMD, %	0.28	0.32	0.48	1.03	1.39	1.71				
Cold-active α-amylase	Supplement level, U/g	68.75	137.50	275.00	550.00	1,100.00	2,200.00	y = -0.0000001x^2^ + 0.0004x + 0.128	<0.01	2,000.00	0.53
	Improved DMD, %	0.15	0.18	0.27	0.35	0.50	0.64				

RSA: Reducing sugar release rate; DMD: Dry matter digestibility.

Quadratic regression equations of supplement level of exogenous carbohydrase individuals and RSA, as well as improved DMD in diets for the broilers aged 1 to 3 wk (Table 4). Based on the data, we calculated the optimal supplement levels of each exogenous carbohydrase for the highest RSA and improved DMD, that 269.65 U/g of glucoamylase, 532.5 U/g pullulanase, 2 U/g maltase, 1385.71 U/g thermostable α-amylase, 269.5 U/g medium temperature α-amylase, and 1965 U/g cold-active α-amylase respectively could improve the RSA of diets to the maximum; and 300 U/g of glucoamylase, 550 U/g pullulanase, 3.2 U/g maltase, 1500 U/g thermostable α-amylase, 285 U/g medium temperature α-amylase, and 2000 U/g cold-active α-amylase respectively could improve the DMD of diets to the maximum.

### Response surface analysis of RSA and improved DMD of broiler diets supplemented with the exogenous carbohydrases

The Box-Behnken design of 6 exogenous carbohydrases and the predicted RSA and improved DMD for 54 experiments were showed in Table 5. Of the 54 experiments, experiment 50 (300 U/g glucoamylase, 550 U/g pullulanase, 2.6 U/g maltase, 1500 U/g thermostable α-amylase, 277.25 U/g medium temperature α-amylase, 1982.5 U/g cold-active α-amylase) and experiment 11 (300 U/g glucoamylase, 541.28 U/g pullulanase, 2.6 U/g maltase, 1500 U/g thermostable α-amylase, 285 U/g medium temperature α-amylase, 1982.5 U/g cold-active α-amylase) produced the maximum amount of RSA (214.19 mg/g) and improved DMD (6.21%), respectively. The lowest amount of RSA (170.73 mg/g) and improved DMD (4.88%) were observed in experiment 8 (284.82 U/g glucoamylase, 532.56 U/g pullulanase, 2 U/g maltase, 1442.86 U/g thermostable α-amylase, 269.5 U/g medium temperature α-amylase, 1982.5 U/g cold-active α-amylase) and experiment 35 (284.82 U/g glucoamylase, 532.56 U/g pullulanase, 2.6 U/g maltase, 1442.86 U/g thermostable α-amylase, 269.5 U/g medium temperature α-amylase, 2000 U/g cold-active α-amylase), respectively.

**Table 5:** Box-Behnken design of exogenous carbohydrases with coded values, predicted RSA and improved DMD.

	Factors							
	A	B	C	D	E	F	Y_1_	Y_2_
Experiment	Glucoamylase, U/g	Pullulanase, U/g	Maltase, U/g	Thermostable α-amylase, U/g	Medium temperature α-amylase, U/g	Cold-active α-amylase, U/g	RSA, mg/g	Improved DMD, %
1	269.65	541.28	2.60	1,500.00	285.00	1,982.50	195.95	5.56
2	269.65	532.56	2.60	1,500.00	277.25	1,982.50	185.42	5.31
3	284.82	541.28	2.60	1,442.86	277.25	1,982.50	200.65	5.81
4	284.82	550.00	3.20	1,442.86	269.50	1,982.50	197.43	5.69
5	284.82	532.56	3.20	1,442.86	285.00	1,982.50	197.54	5.56
6	269.65	541.28	3.20	1,442.86	277.25	2,000.00	193.59	5.48
7	284.82	541.28	3.20	1,385.71	277.25	2,000.00	194.03	5.56
8	284.82	532.56	2.00	1,442.86	269.50	1,982.50	170.73	4.89
9	269.65	541.28	3.20	1,442.86	277.25	1,965.00	186.07	5.33
10	300.00	532.56	2.60	1,500.00	277.25	1,982.50	198.85	5.79
11	300.00	541.28	2.60	1,500.00	285.00	1,982.50	209.51	6.21
12	269.65	541.28	2.00	1,442.86	277.25	1,965.00	175.28	4.99
13	284.82	541.28	2.00	1,385.71	277.25	1,965.00	178.46	5.11
14	300.00	541.28	2.00	1,442.86	277.25	1,965.00	190.08	5.39
15	300.00	532.56	2.60	1,385.71	277.25	1,982.50	186.27	5.44
16	284.82	550.00	2.00	1,442.86	269.50	1,982.50	184.92	5.22
17	284.82	550.00	2.60	1,442.86	269.50	1,965.00	186.08	5.39
18	284.82	532.56	2.60	1,442.86	285.00	2,000.00	191.95	5.48
19	284.82	541.28	2.60	1,442.86	277.25	1,982.50	200.70	5.80
20	269.65	541.28	2.60	1,500.00	269.50	1,982.50	183.11	5.19
21	284.82	541.28	2.60	1,442.86	277.25	1,982.50	200.59	5.83
22	284.82	550.00	2.60	1,442.86	269.50	2,000.00	191.54	5.59
23	284.82	532.56	2.60	1,442.86	269.50	1,965.00	175.21	4.98
24	269.65	550.00	2.60	1,500.00	277.25	1,982.50	194.92	5.58
25	284.82	532.56	2.00	1,442.86	285.00	1,982.50	184.77	5.55
26	284.82	541.28	2.60	1,442.86	277.25	1,982.50	200.54	5.77
27	269.65	532.56	2.60	1,385.71	277.25	1,982.50	175.37	5.03
28	300.00	541.28	2.00	1,442.86	277.25	2,000.00	194.34	5.81
29	284.82	550.00	2.60	1,442.86	285.00	1,965.00	194.07	5.56
30	300.00	541.28	2.60	1,385.71	269.50	1,982.50	187.33	5.31
31	284.82	541.28	2.00	1,500.00	277.25	1,965.00	185.45	5.31
32	300.00	541.28	2.60	1,500.00	269.50	1,982.50	202.29	5.88
33	284.82	541.28	2.00	1,385.71	277.25	2,000.00	174.41	5.99
34	284.82	541.28	3.20	1,500.00	277.25	2,000.00	206.97	5.88
35	284.82	532.56	2.60	1,442.86	269.50	2,000.00	172.39	4.88
36	269.65	541.28	2.60	1,385.71	285.00	1,982.50	190.97	5.47
37	269.65	541.28	2.60	1,385.71	269.50	1,982.50	170.75	4.83
38	300.00	541.28	3.20	1,442.86	277.25	1,965.00	195.87	5.61
39	300.00	541.28	2.60	1,385.71	285.00	1,982.50	201.94	5.73
40	284.82	541.28	3.20	1,500.00	277.25	1,965.00	191.18	6.19
41	300.00	541.28	3.20	1,442.86	277.25	2,000.00	208.85	5.98
42	300.00	550.00	2.60	1,385.71	277.25	1,982.50	204.25	5.85
43	284.82	541.28	2.60	1,442.86	277.25	1,982.50	200.76	5.65
44	269.65	550.00	2.60	1,385.71	277.25	1,982.50	187.55	5.37
45	284.82	550.00	2.60	1,442.86	285.00	2,000.00	208.52	5.97
46	284.82	532.56	2.60	1,442.86	285.00	1,965.00	185.65	5.32
47	284.82	550.00	3.20	1,442.86	285.00	1,982.50	210.88	6.19
48	284.82	541.28	2.60	1,442.86	277.25	1,982.50	200.61	5.79
49	284.82	532.56	3.20	1,442.86	269.50	1,982.50	181.62	5.18
50	300.00	550.00	2.60	1,500.00	277.25	1,982.50	214.19	6.14
51	269.65	541.28	2.00	1,442.86	277.25	2,000.00	174.24	5.05
52	284.82	541.28	2.00	1,500.00	277.25	2,000.00	192.51	5.48
53	284.82	541.28	3.20	1,385.71	277.25	1,965.00	189.34	5.33
54	284.82	550.00	2.00	1,442.86	285.00	1,982.50	196.41	5.58

RSA: Reducing sugar release rate; DMD: Dry matter digestibility.

Table 6 showed the results of the ANOVA of the quadratic polynomial model for optimization of the exogenous carbohydrases zymogram for optimal RSA and improved DMD in diets for broilers aged 1 to 3 wk. The models were highly significant (*P* < 0.0001), and the “Lack of Fit” of the models were not significant (*P* > 0.05), which demonstrated the RSA and improved DMD were proper parameters representing the starch digestibility of the broilers, and the models were applicable to the optimization of exogenous carbohydrases for higher RSA and improved DMD in diets for the broilers.

**Table 6:** ANOVA for the quadratic polynomial model for optimization of the exogenous carbohydrases zymogram for optimal RS and DMD in diets for broiler aged 1 to 3 wk.

Source	Sum of Squares		Degree of freedom		Mean Square		*F—*Value		*P—*value	
	RSA	Improved DMD	RSA	Improved DMD	RSA	Improved DMD	RSA	Improved DMD	RSA	Improved DMD
Model	6425.04	6.310	27	27	237.96	0.230	99,115.46	27.37	< 0.0001	< 0.0001
A	1358.26	1.480	1	1	1358.26	1.480	566,000.00	172.78	< 0.0001	< 0.0001
B	1134.24	0.930	1	1	1134.24	0.930	472,000.00	108.73	< 0.0001	< 0.0001
C	959.76	0.540	1	1	959.76	0.540	400,000.00	63.6	< 0.0001	< 0.0001
D	596.80	0.510	1	1	596.8	0.510	249,000.00	59.79	< 0.0001	< 0.0001
E	1131.08	1.110	1	1	1131.08	1.110	471,000.00	129.44	< 0.0001	< 0.0001
F	207.68	0.290	1	1	207.68	0.290	86,502.26	34.01	< 0.0001	< 0.0001
AB	16.94	0.003	1	1	16.94	0.003	7,054.16	0.33	< 0.0001	0.5709
AC	12.10	0.018	1	1	12.1	0.018	5,041.15	2.11	< 0.0001	0.1579
AD	6.62	0.035	1	1	6.62	0.035	2,756.39	4.12	< 0.0001	0.0528
AE	15.76	0.008	1	1	15.76	0.008	6,565.97	0.99	< 0.0001	0.3290
AF	14.47	0.042	1	1	14.47	0.042	6,027.87	4.93	< 0.0001	0.0354
BC	1.38	0.076	1	1	1.38	0.076	573.87	8.91	< 0.0001	0.0061
BD	3.54	0.002	1	1	3.54	0.002	1,473.54	0.25	< 0.0001	0.6231
BE	6.31	0.020	1	1	6.31	0.020	2,629.31	2.38	< 0.0001	0.1351
BF	33.74	0.038	1	1	33.74	0.038	14,054.47	4.43	< 0.0001	0.0452
CD	13.29	0.280	1	1	13.29	0.280	5,534.22	32.51	< 0.0001	< 0.0001
CE	1.84	0.002	1	1	1.84	0.002	767.72	0.29	< 0.0001	0.5967
CF	75.47	0.074	1	1	75.47	0.074	31,435.4	8.7	< 0.0001	0.0067
DE	27.27	0.016	1	1	27.27	0.016	11,357.96	1.9	< 0.0001	0.1801
DF	61.66	0.200	1	1	61.66	0.200	25,682.45	22.88	< 0.0001	< 0.0001
EF	41.00	0.028	1	1	41.00	0.028	17,075.61	3.23	< 0.0001	0.0837
A^2^	31.29	0.180	1	1	31.29	0.180	13,030.78	20.75	< 0.0001	0.0001
B^2^	95.99	0.100	1	1	95.99	0.100	39,980.36	12.05	< 0.0001	0.0018
C^2^	117.04	0.027	1	1	117.04	0.027	48,746.84	3.16	< 0.0001	0.087
D^2^	63.79	0.004	1	1	63.79	0.004	26,568.03	0.48	< 0.0001	0.4937
E^2^	139.00	0.210	1	1	139.00	0.210	57,894.99	24.04	< 0.0001	< 0.0001
F^2^	338.23	0.190	1	1	338.23	0.190	141,000.00	22.78	< 0.0001	< 0.0001
Residual	0.06	0.220	26	26	0.0024	0.009				
Lack of Fit	0.03	0.200	21	21	0.0015	0.010	0.23	2.31	0.9923	0.1796
Pure Error	0.03	0.021	5	5	0.0063	0.004				
Corr. Total	6425.10	6.530	53	53						
*R*_*RSA*_^2^ = 1.0000										
*R*_*Improved DMD*_^2^ = 0.9660										

**Fig 1:**
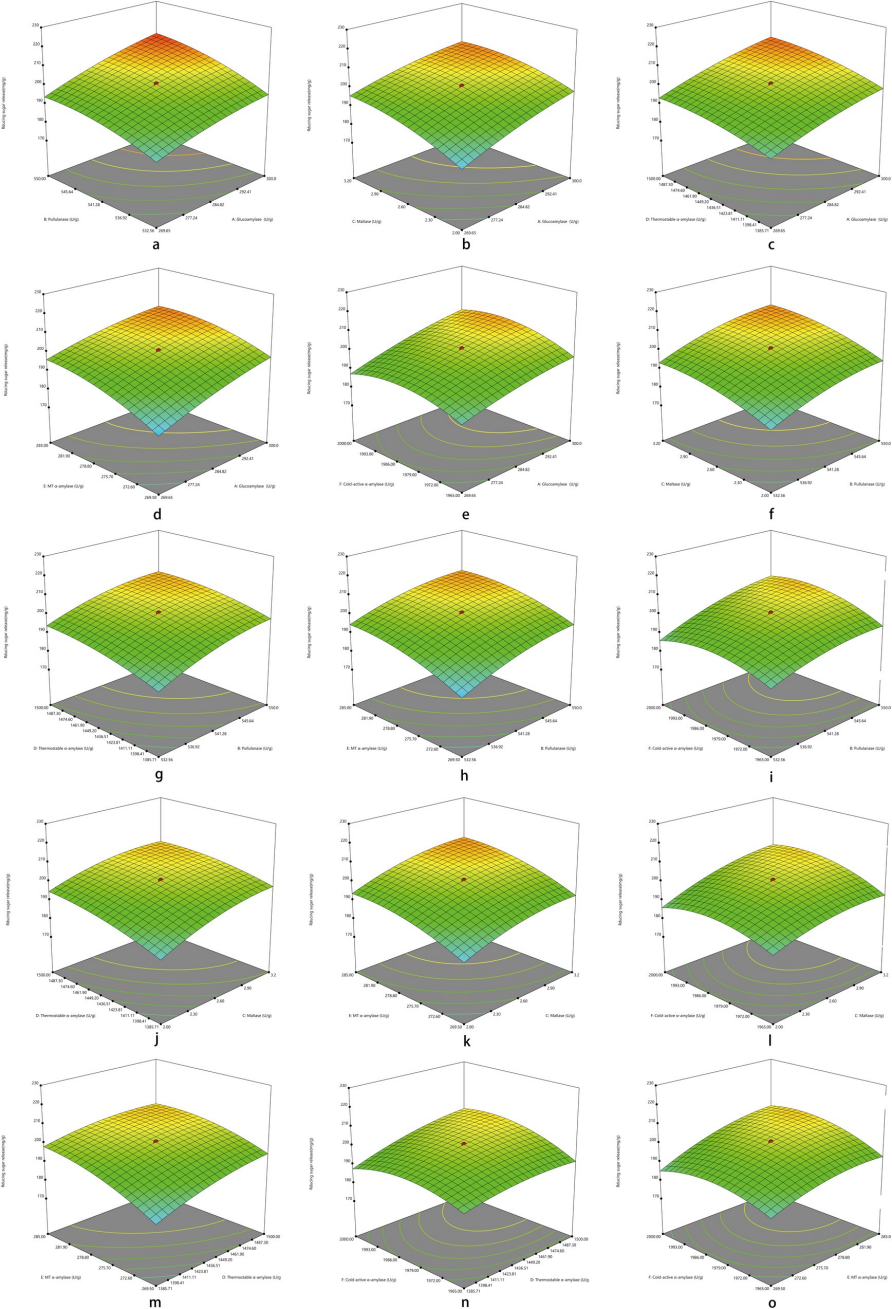
Respond surface plots for the significant interactions of exogenous carborhydrases on the RSA in diets for broilers aged 1 to 3 wk. (a) to (o) representing interactions of AB, AC, AD, AE, AF, BC, BD, BE, BF, CD, CE, CF, DE, DF, and EF, respectively.

**Fig 2:**
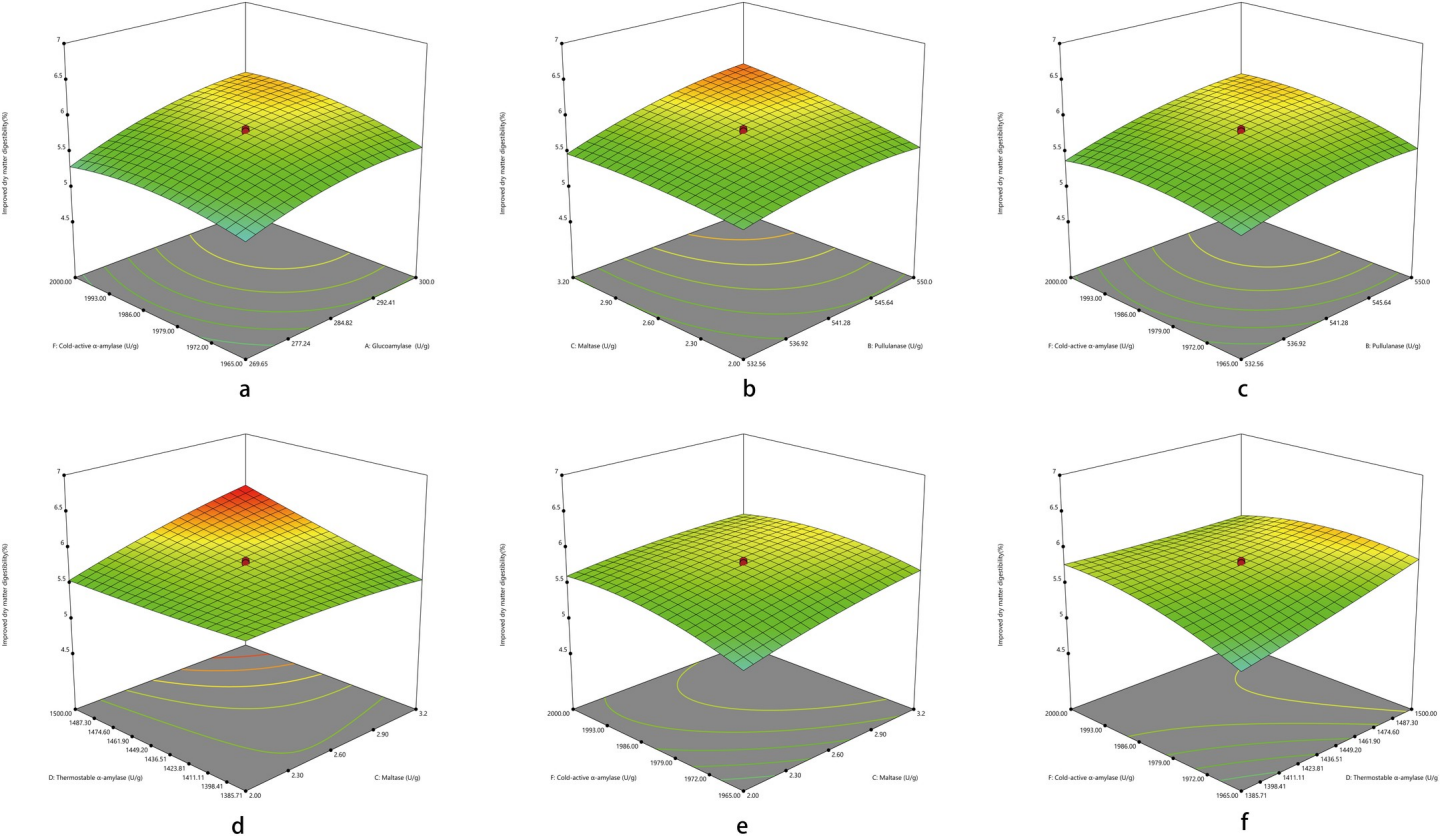
Respond surface plots for the significant interactions of exogenous carborhydrases on the improved DMD in diets for broilers aged 1 to 3 wk. (a) to (f) representing interactions of AF, BC, BF, CD, CF, and DF, respectively.

Three-dimensional images of the response surface were generated to demonstrate the significant interactions among the tested carborhydrases and the RSA as well as improved DMD in diets for broilers aged 1 to 3 wk. As showed in Table 6, the effects of all the interactions among tested exogenous carborhydrases on RSA were significant (*P* < 0.01), which were demonstrated in Fig 1. Regarding to improved DMD, only interactions between A and F (*P* = 0.0354), B and C (*P* = 0.0061), B and F (*P* = 0.0452), C and D (*P* < 0.0001), C and F (*P* = 0.0067), D and F (*P* < 0.0001) showed significant effect, which were demonstrated in Fig 2.

As seen in Eqs 4 and 5, the results of the predicted responses of RSA (Y_1_) and improved DMD (Y_2_) in diets supplemented with exogenous carbohydrases according to previously stated second-order polynomial Eq 3 are as follows, respectively:

$$Y_{1}=200.64+7.52A+6.89B+6.32C+4.99D+6.87E+2.94F+1.46AB-1.23AC+0.64AD-1.4AE+1.34AF+0.42BC-0.66BD-0.63BE+2.05BF-1.29CD+0.48CE+2.17CF-1.85DE+2.78DF+2.26EF-1.74A^{2}-3.05B^{2}-3.37C^{2}-2.49D^{2}-3.68E^{2}-5.73F^{2}$$ (4)

$$Y_{2}=5.77+0.25A+0.2B+0.15C+0.15D+0.21E+0.11F+0.019AB-0.047AC+0.047AD-0.032AE+0.073AF+0.098BC-0.016BD-0.036BE+0.069BF+0.19CD-0.018CE-0.068CF-0.045DE-0.16DF+0.059EF-0.13A^{2}-0.1B^{2}-0.051C^{2}+0.02D^{2}-0.014E^{2}-0.14F^{2}$$ (5)

According to the results of response surface analysis, the optimum zymogram were obtained as follows: glucoamylase, 297.39 U/g; pullulanase, 549.72 U/g; maltase, 3.01 U/g; thermostable α-amylase, 1,455.73 U/g; medium temperature α-amylase, 278.64 U/g; cold-active α-amylase, 1,985.97 U/g. With the optimum zymogram, the predicted RSA and improved DMD in diets for broilers aged 1 to 3 wk were 215.98 mg/g, and 6.23%, respectively.

### *In vitro* simulated digestion study of RSA and improved DMD of broiler diets supplemented with the optimum zymogram

*In vitro* simulated digestion experiments were carried out to verify the predicted RSA and improved DMD in diets supplemented with the optimum zymogram. The experiments were repeated five times and the mean experimental RSA and improved DMD were 219.26 mg/g and 6.31%, respectively, which were compatible with the predicted RSA and improved DMD values.

## Discussion

Although broilers have high innate capacity to digest dietary starch, it can be limited by several factors such as inadequacies in endogenous starch digestion enzymes, the complicated nature of the starch crystals, and difficulties around extraction of glucose from the lumen via Na-dependent transport systems [17]. Supplementation of exogenous carbohydrases is a common strategy to improve the digestibility of starch in poultry diets, especially for younger birds with less developed gastrointestinal tract and microbiome involved. Many studies proved that exogenous carbohydrasses in diets improved the body weight, and decreased the feed to meat ratio in broilers [18–20]. Gracia et al. [11] reported that supplement of exogenous α-amylase significantly improved the AMEn, starch digestion rate, and growth performance, and decreased the pancreas weight in broilers. Other than directly participated in the process of starch digestion, exogenous carbohydrase within certain rate ranges could beneficially influence the secretions and activities of some endogenous digestion enzymes, such as protease, trypsin in the anterior intestine [19], which may be another reason to explain the benefits of supplement of exogenous carbohydrases in broiler diets. However, high rate of exogenous carbohydrase (2250 mg/kg mentioned in the paper) may demonstrate reverse effects [19].

Starch is complex carbohydrate reserves and can be deformed and converted into many substances, such as maltose, glucose, fructose, organic acids, amino acids, etc. [21]. Multiple carbohydrases are required to be participated in the process of starch digestion. Glucoamylase is an exo-acting enzyme that mainly hydrolyzes α–1, 4–glycosidic linkages from the non-reducing ends of starch chains, which leads to the production of glucose [22]. Pullulanase can specifically hydrolyze α–1, 6-glycosidic linkages in starch and oligosaccharides [23]. In the small intestine, maltase can catalyze the digestion of maltose, which were metabolite from disaccharidases [24]. As the widest produced starch digestion enzyme, α-amylase is found in almost all living organism and it is one of the most vital enzymes in starchy substrates digestion. It catalyzes the α–1, 4-glycosidic linkages in starch, glycogen and related polysaccharides and oligosaccharides and release reducing groups in α-configuration, for example maltose and malto-oligasaccharides [25]. Also, as the most important industrial enzymes, α-amylases are expected to be used from high to low temperatures, to prevent starch granules precipitation in the hydrolysis process, facilitate the downstream refining processes, and reduce costs [26,27]. Therefore, α-amylases with adoptions to high, medium, and low temperatures were invented via genetic engineering and bio-engineering. The maximum utilization of starch needs the combination of carbohydrases targeting specific metabolites under certain environmental conditions, instead of the effects of individuals [28]. In the present study, we used Box Behnken design to predict the optimistic combination of glucoamylase, pullulanase, maltase, thermostable α-amylase, medium temperature α-amylase, and cold-active α-amylase for starch digestion, represented by RSA and improved DMD, in diets for broilers aged 1 to 3 wk. It was showed that with 297.39 U/g glucoamylase, 549.72 U/g pullulanase, 3.01 U/g maltase, 1,455.73 U/g thermostable α-amylase, 278.64 U/g medium temperature α-amylase, and 1,985.97 U/g cold-active α-amylase supplemented in broiler diet, the RSA and improved DMD reached the maximum of 215.98 mg/g and 6.23% respectively. Moreover, we verify the predicted RSA and improved DMD, and found out that the mean experimental RSA and improved DMD were 219.26 mg/g and 6.31% respectively, whose error to the predicted optimistic RSA and DMD were 1.05% and 1.02%. It showed that the predicted combination of 6 carbohydrases were compatible with the actual optimal zymogram. These results were consisted with previous study that combination of multiple exogenous carbohydrases could improve the starch digestibility of broiler diets [29].

It was highly important for the animal nutritionists to evaluate the effects of exogenous enzymes in fast and accurate methods to help the optimizations of animal feed. However, with the method of *in vivo* animal experiments, it was not practical to use for the critical limitations, for example long-time consuming and variations in animal conditions. Additionally, the effects of exogenous enzymes were influenced by other factors such as diet ingredients, enzymes origins, and supplement rates, which made the results even more inconsistent. Many attempts were made by researchers using method of *in vitro* simulated gastrointestinal digestion. Malathi et al. [30] proved that the methodology of *in vitro* experiment with simulated gastrointestinal fluid were applicable to evaluate the effectiveness and stability of exogenous enzymes in poultry. In the present study, we used the SDS-Ⅲ to simulate the digestion process of diets in the stomach and intestine, evaluated the effects of 6 exogenous carbohydrases on the starch digestibility in diets of broiler aged 1 to 3 wk. We found that there were significant quadratic relationships between the supplement ratio of the individual exogenous carbohydrase and the parameters associated with starch digestibility, which were consistent previous finding that dietary supplementation of exogenous carbohydrases needed to be in proper level for the beneficial effects [19].

## Conclusion

In conclusion, by using *in vitro* simulated gastrointestinal digestion and response surface method, we predicted the optimal zymogram of 6 carbohydrases supplemented in corn-soybean based diet for broilers aged 1 to 3 wk, which were 297.39 U/g glucoamylase, 549.72 U/g pullulanase, 3.01 U/g maltase, 1,455.73 U/g thermostable α-amylase, 278.64 U/g medium temperature α-amylase, and 1,985.97 U/g cold-active α-amylase, to increases 215.98 mg/g reduced sugar release amount and 6.23% dry matter digestibility. However, the actual effects of the combination of experimented exogenous carbohydrases on the starch digestion of broiler diets still needs further works before utilizing in the feed formula. For example, *in vivo* experiment with the predicted optimistic zymogram of 6 carbohydrases are needed. And more parameters representing the level of starch digestion can be considered in future study.
